# Oligomeric Proanthocyanidins Reverse Lenvatinib Resistance in Hepatocellular Carcinoma Through ITGA3-Mediated Pathway

**DOI:** 10.3390/ph18091361

**Published:** 2025-09-12

**Authors:** Takayuki Noma, Yuan Li, Yuma Wada, Yuji Morine, Tetsuya Ikemoto, Yu Saito, Shinichiro Yamada, Hiroki Teraoku, Mitsuo Shimada, Ajay Goel

**Affiliations:** 1 Department of Molecular Diagnostics and Experimental Therapeutics, Beckman Research Institute of City of Hope, Biomedical Research Center, Monrovia, CA 91016, USA; tnoma@coh.org (T.N.); yuali@coh.org (Y.L.); 2 Department of Surgery, Tokushima University, Tokushima 779-1510, Japan; wada.yuuma@tokushima-u.ac.jp (Y.W.); ymorine@tokushima-u.ac.jp (Y.M.); ikemoto.tetsuya@tokushima-u.ac.jp (T.I.); saito.yu.1001@tokushima-u.ac.jp (Y.S.); yamada.shinichirou@tokushima-u.ac.jp (S.Y.); teraoku.hiroki@tokushima-u.ac.jp (H.T.); mitsuo.shimada@tokushima-u.ac.jp (M.S.); 3 Department of Clinical Laboratory, Yangpu Hospital, Tongji University School of Medicine, Shanghai 200090, China; 4 City of Hope Comprehensive Cancer Center, Duarte, CA 91010, USA

**Keywords:** hepatocellular carcinoma, oligomeric proanthocyanidins, lenvatinib resistance, synergistic effect, anoikis, ITGA3

## Abstract

**Background**: Oligomeric proanthocyanidins (OPCs) are natural polyphenolic compounds with strong antitumor properties and have gained attention as potential agents to overcome drug resistance. Hepatocellular carcinoma (HCC) remains a major cause of cancer deaths worldwide, and although Lenvatinib is widely used, its effectiveness is limited by acquired resistance. This study explores the potential of OPCs to overcome Lenvatinib resistance in HCC. **Methods**: To evaluate the potential of OPCs to overcome Lenvatinib resistance in HCC, we established Lenvatinib-resistant Huh-7 and PLC-PRF-5 cell lines and conducted systematic cell culture experiments to assess their antitumor effects. Furthermore, genome-wide transcriptomic profiling, network pharmacology approaches, and pathway enrichment analysis were performed to identify resistance-associated signaling pathways that could serve as therapeutic targets. **Results**: The combination of OPCs and Lenvatinib demonstrated a significant synergistic anti-proliferative effect in resistant hepatocellular carcinoma cells, with the most synergistic dose combinations showing Bliss synergy scores exceeding 10. Transcriptomic profiling revealed that the adhesion molecule ITGA3 is a key factor in Lenvatinib resistance and contributes to the acquisition of anoikis resistance. The combination treatment suppressed ITGA3–EGFR–AKT signaling, restored anoikis sensitivity, significantly reduced spheroid formation (fold change = 0.10–0.12; *p* < 0.001), and markedly increased apoptosis (fold change = 2.7–5.0; *p* < 0.001). **Conclusions**: This study is the first to demonstrate that OPCs can overcome chemotherapy resistance by targeting the integrin pathway, providing scientific evidence for their potential use as an adjunctive therapy for chemotherapy-resistant HCC.

## 1. Introduction

Hepatocellular carcinoma (HCC) is the major type of primary liver cancer, with approximately 800,000 cases annually, ranking as the third leading cause of cancer-related deaths [1]. More than half of HCC patients are diagnosed at an advanced stage, where systemic therapy remains the primary treatment option. Sorafenib and Lenvatinib, both multi-tyrosine kinase inhibitors (TKIs), were the first systemic agents introduced for the treatment of advanced HCC. Sorafenib was approved as first-line therapy in 2007 [2,3], and in 2018, Lenvatinib was approved as an alternative following the REFLECT trial, which demonstrated its non-inferiority to Sorafenib [4]. More recently, immune checkpoint inhibitors (ICIs), particularly PD-1 inhibitors, have rapidly transformed the treatment landscape for HCC, with the IMbrave150 trial establishing atezolizumab plus bevacizumab as an effective option [5,6]. On the other hand, Lenvatinib has still been reported to be effective for non-viral HCC, including cases related to metabolic disorders and diabetes-associated NASH, which are expected to increase in the future [7,8,9]. Therefore, its clinical use continues to be relevant and sustained. However, the overall response rate of Lenvatinib remains limited at around 24%, and complex resistance mechanisms, including Epidermal growth factor receptor (EGFR) pathway activation, RNA modifications, autophagy, and ferroptosis, have been reported to contribute to this resistance [10,11,12,13]. In recent years, to address Lenvatinib resistance, combination strategies with other molecular targeted agents or ICIs have been regarded as promising approaches [11,14,15]. Nevertheless, challenges such as increased toxicity and high cost remain. Therefore, developing cost-effective and less toxic combination therapies to overcome resistance remains critical for improving HCC treatment.

In this context, several naturally derived plant compounds have garnered increasing attention from researchers and clinicians due to their multi-targeted mechanisms and safer therapeutic profiles compared to conventional chemotherapies and molecular targeted therapies [16,17,18]. Oligomeric proanthocyanidins (OPCs), a class of polyphenolic compounds, have received significant attention in recent years due to their potential antioxidant and antitumor properties. OPCs are abundantly found in grape seeds and are present in a wide range of plants with purple or red pigmentation. Previous research conducted by our group has revealed that the antitumor activity of OPCs is mediated through several key pathways, including the PI3K-Akt signaling, ferroptosis [19], DNA replication and cell cycle regulation [20], ABC transporters [21], and the Hippo signaling [22]. Furthermore, recent studies have demonstrated the synergistic and cooperative effects of OPCs with other natural compounds and therapies [22]. Based on this evidence, we hypothesize that OPCs might synergistically regulate multiple cancer cell signaling pathways, exhibiting potent antitumor activity in HCC, and potentially overcoming chemotherapy resistance.

In this study, we systematically evaluated for the first time the antitumor effects of OPCs using network pharmacology approaches in HCC using two different HCC cell lines. Additionally, we performed genome-wide transcriptomic profiling analysis to identify the molecular mechanism(s) and key pathways associated with Lenvatinib resistance in HCC. Finally, we validated the mechanism by which OPCs overcome Lenvatinib resistance.

## 2. Results

### 2.1. Establishment of Lenvatinib-Resistant HCC Cell Lines and Evaluation of OPCs-Mediated Synergistic Effects

**Figure 1:**
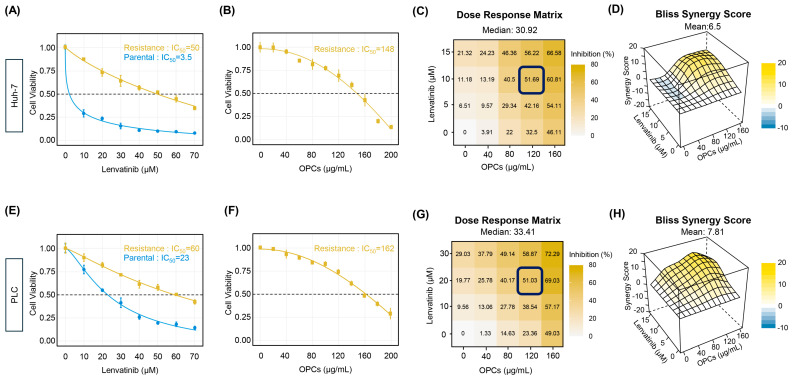
Establishment of Lenvatinib-Resistant HCC Cell Lines and Synergistic Effects of OPCs and Lenvatinib. (**A**,**E**) Dose–response curves of Lenvatinib in parental (pHuh-7 and pPLC) and resistant (rHuh-7 and rPLC) HCC cell lines. The IC_50_ of Lenvatinib increased markedly in resistant cells: rHuh-7 showed a >10-fold increase compared to pHuh-7 (50.3 μM vs. 3.5 μM), and rPLC showed a 2-fold increase compared to pPLC (59.7 μM vs. 23.1 μM). (**B**,**F**) Dose–response curves of OPCs in Lenvatinib-resistant HCC cell lines (rHuh-7 and rPLC). OPCs reduced cell viability in a dose-dependent manner, with IC_50_ values of 147.9 μg/mL for rHuh-7 and 162.0 μg/mL for rPLC. (**C**,**G**) Dose–response matrices showing the percentage reduction in cell viability of rHuh-7 and rPLC cells treated with different combinations of Lenvatinib and OPCs. In rHuh-7 cells, the combination of 10 μM Lenvatinib with 120 μg/mL OPCs reduced cell viability by 51.7%. In rPLC cells, the combination of 20 μM Lenvatinib with 120 μg/mL OPCs reduced viability by 51.0%. (**D**,**H**) Bliss synergy scores for the combinations of Lenvatinib and OPCs in rHuh-7 and rPLC cells. The most synergistic combinations achieved scores of 12.5 in rHuh-7 and 11.6 in rPLC, indicating robust synergistic effects in both cell lines.

To investigate whether OPCs can help overcome Lenvatinib resistance in HCC, we established Lenvatinib-resistant Huh-7 cells (rHuh-7) and Lenvatinib-resistant PLC-PRF-5 cells (rPLC) by gradually increasing the concentration of Lenvatinib over several months in parental Huh-7 cells (pHuh-7) and PLC-PRF-5 cells (pPLC). Notably, the half-maximal inhibitory concentration (IC_50_) of Lenvatinib was significantly higher in the resistant cell lines compared to their respective parental controls, with rHuh-7 exhibiting a more than 10-fold increase in resistance vs. pHuh-7 (IC_50_: 50.3 μM vs. 3.5 μM; Figure 1A), and rPLC showing a 2-fold increase in resistance vs. pPLC cells (IC_50_: 59.7 μM vs. 23.1 μM; Figure 1E).

Next, we assessed whether OPCs could exert antiproliferative effects in HCC cell lines. OPCs reduced cell viability in a dose-dependent manner, with IC_50_ values of 147.9 μg/mL for rHuh-7 (Figure 1B) and 162.0 μg/mL for rPLC (Figure 1F). Since the goal was to evaluate whether OPCs can overcome Lenvatinib resistance in HCC cells, we planned combinatorial treatments with varying concentrations of Lenvatinib and OPCs in Lenvatinib-resistant HCC cell lines to decipher the synergistic effects of these compounds. Criteria were established to identify the optimal dose ratio of Lenvatinib and OPCs in combination therapy (dose response inhibited by >50% and the highest Bliss synergy score). The concentration of OPCs was set at (0, 40, 80, 120, 160 μg/mL), considering the IC_50_ for OPCs in each cell line. On the other hand, the concentration of Lenvatinib was set to (0, 5, 10, 15 μM) for the rHuh-7 and (0, 10, 20, 30 μM) for the rPLC, considering that the IC_50_ of Lenvatinib in the parental cell lines was different, at 3.5 μM and 23.1 μM, respectively. In rHuh-7 cells, the combination of 10 μM Lenvatinib with 120 μg/mL OPCs inhibited cell growth by 51.7%, compared with 11.2% for Lenvatinib alone and 32.5% for OPCs alone, with a Bliss synergy score of 12.5 (Figure 1C,G). In rPLC cells, the combination of 20 μM Lenvatinib with 120 μg/mL OPCs inhibited growth by 51.0%, compared with 19.8% and 23.4% for single treatments, with a Bliss synergy score of 11.6 (Figure 1D,H). Accordingly, we used 10 μM Lenvatinib plus 120 μg/mL OPCs for rHuh-7, and 20 μM Lenvatinib plus 120 μg/mL OPCs for rPLC in subsequent experiments. These results indicate that OPCs exhibit apparent antitumor activity and demonstrate a significant synergistic effect with Lenvatinib in resistant HCC cells, providing an optimal basis for subsequent combination experiments.

### 2.2. Evaluation of the Effects of Lenvatinib and OPCs on Cell Migration and Invasion

**Figure 2:**
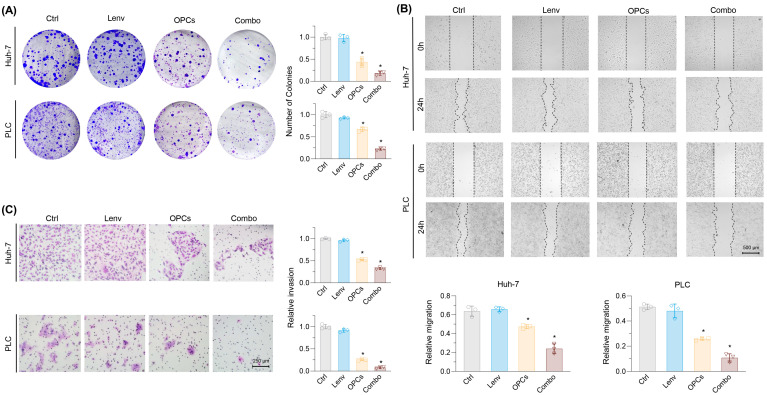
Evaluation of the Effects of Lenvatinib and OPCs on Colony Formation, Migration, and Invasion in rHuh-7 and rPLC Cells. (**A**) Colony formation in Lenvatinib-resistant cell lines (rHuh-7, rPLC) treated with Lenvatinib (Lenv), OPCs, or the combination. Lenvatinib alone had little effect, while OPCs significantly reduced colony formation. The combination treatment further decreased colony formation in both cell lines. (**B**) Wound healing assay showing the impact on cell migration. Lenvatinib alone had no effect, but the combination with OPCs significantly reduced migration in both rHuh-7 and rPLC. (**C**) Invasion assay results. Lenvatinib alone did not affect invasion, but combination treatment with OPCs significantly inhibited invasion in both cell lines. Data represent mean ± SD (* *p* < 0.05 vs. control).

We next assessed the effects of Lenvatinib and OPCs on colony formation, migration, and invasion in Lenvatinib-resistant HCC cells. Lenvatinib alone had a minimal impact on colony formation in both rHuh-7 and rPLC, whereas OPCs significantly reduced colony-forming ability. Notably, the combination treatment further suppressed colony formation by 82.0% in rHuh-7 and 77.8% in rPLC (*p* < 0.01; Figure 2A). Wound healing assays revealed no difference between control and Lenvatinib-treated cells. In contrast, combination therapy significantly impaired cell migration, as indicated by a log2 fold change (FC) of 0.37 in rHuh-7 and 0.21 in rPLC (*p* < 0.01; Figure 2B). Similarly, in invasion assays, Lenvatinib alone had little effect, while the combination with OPCs significantly reduced invasion (rHuh-7: 0.32, rPLC: 0.08 relative to control; *p* < 0.01; Figure 2C). Together, these results demonstrate that the combination of OPCs and Lenvatinib significantly suppresses malignant potential in Lenvatinib-resistant HCC cells.

### 2.3. Combination of OPCs and Lenvatinib Induces Apoptosis and Suppresses Cancer Stemness in Resistance HCC Cell Lines

**Figure 3:**
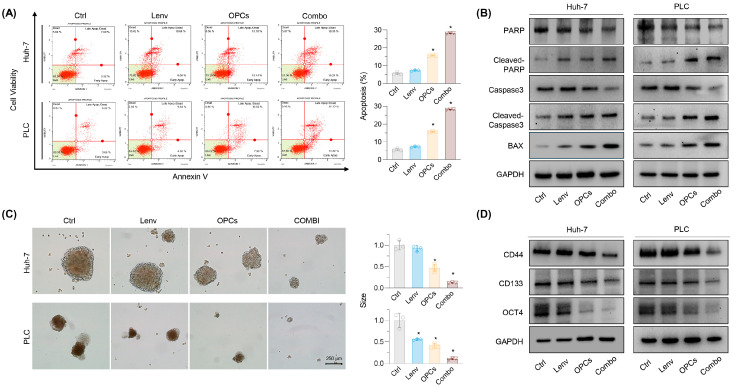
OPCs Induce Apoptosis and Suppress Cancer Stemness in Lenvatinib-Resistant HCC Cells. (**A**) Annexin V assay showing the effects of Lenvatinib (Lenv), OPCs, and their combination on apoptosis in Lenvatinib-resistant cell lines (rHuh-7, rPLC). Combination therapy significantly increased apoptosis in both cell lines compared to control and Lenv alone. (**B**) Western blot analysis of apoptosis-related proteins (PARP, cleaved-PARP, Caspase 3, cleaved-Caspase 3, BAX) in rHuh-7 and rPLC after treatment. Combination therapy demonstrated enhanced expression of apoptosis markers compared to monotherapy. (**C**) Spheroid formation assay in rHuh-7 and rPLC. OPCs and combination treatments significantly reduced the size of spheroids. (**D**) Western blot analysis of cancer stem cell markers (Nanog, CD44, OCT4) in rHuh-7 and rPLC. The combination of OPCs and Lenvatinib significantly reduced stem cell marker expression compared to control and Lenv alone. Data represent mean ± SD (* *p* < 0.05 vs. control).

To evaluate the apoptotic modulation induced by Lenvatinib and OPCs in HCC cell lines, we performed an Annexin V binding assay. The baseline viable cell rates were 94.3% for rHuh-7 and 89.6% for rPLC. As expected, treatment with Lenvatinib did not affect the viability of the resistant cell lines (rHuh-7: 92.5%, rPLC: 88.9%; Figure 3A). In contrast, treatment with OPCs alone and in combination with Lenvatinib significantly reduced the viable cell rates in both rHuh-7 (OPCs: 84.1%, combination: 71.0%) and rPLC (OPCs: 81.2%, combination: 65.9%; Figure 3A). Furthermore, approximately 25% of the cells underwent apoptosis following the combination therapy with OPCs and Lenvatinib. In rHuh-7, the apoptotic cell rates were as follows: control vs. Lenvatinib vs. OPCs vs. combination = 5.6% vs. 7.3% vs. 15.8% vs. 28.2%, respectively, with significant differences observed between the control and OPCs (*p* < 0.01) and between the control and combination treatment (*p* < 0.01). Similarly, in rPLC, the apoptotic cell rates were as follows: control vs. Lenvatinib vs. OPCs vs. combination, 9.0% vs. 10.7% vs. 17.8% vs. 24.4%, respectively, with significant differences between the control and OPCs (*p* < 0.01) and between the control and combination treatment (*p* < 0.01; Figure 3A). In addition, we examined the expression of apoptosis-related proteins to gain further insight into this cell death mechanism. Western blot analysis revealed increased levels of scleaved PARP, cleaved caspase 3, and BAX, supporting that combination therapy is more effective in inducing apoptosis than monotherapy. These data suggest that the combination of Lenvatinib and OPCs induces apoptosis in HCC cells (Figure 3B). These observations provide clear evidence that the combined use of OPCs and Lenvatinib enhances apoptosis in Lenvatinib-resistant HCC cells.

Next, to determine whether OPCs also affect cancer stemness, we performed spheroid formation assays with cancer spheroids developed from two Lenvatinib-resistant cells. Spheroid size was assessed after the end of treatment; OPCs treatment and Lenvatinib significantly reduced the number and size of spheroids (Figure 3C). In rHuh-7, spheroid size was reduced significantly by OPCs (FC = 0.47; *p* < 0.01), and these effects were even more pronounced by the combination treatment (FC = 0.12; *p* < 0.01). Similar effects were observed in rPLC (OPCs: FC = 0.41; combination: FC = 0.10; *p* < 0.01). Protein expression of the cancer stem markers CD44 and CD133, as well as OCT4, was also assessed in various cancers, including HCC and OPCs. The combination treatment significantly reduced the expression of both markers compared to untreated controls and cells treated with Lenvatinib. The combination of OPCs and Lenvatinib was the most potent inhibitor of protein expression of both stemness markers (Figure 3D). These findings from the cancer spheroid assay highlight that OPCs influence cancer stemness in overcoming Lenvatinib resistance. Overall, our findings demonstrate that OPCs, especially when combined with Lenvatinib, effectively reduce cancer stemness in resistant HCC cells.

### 2.4. Transcriptome Profiling Identified Involved Pathways and Genes That Could Be Targeted to Overcome Lenvatinib Resistance

**Figure 4:**
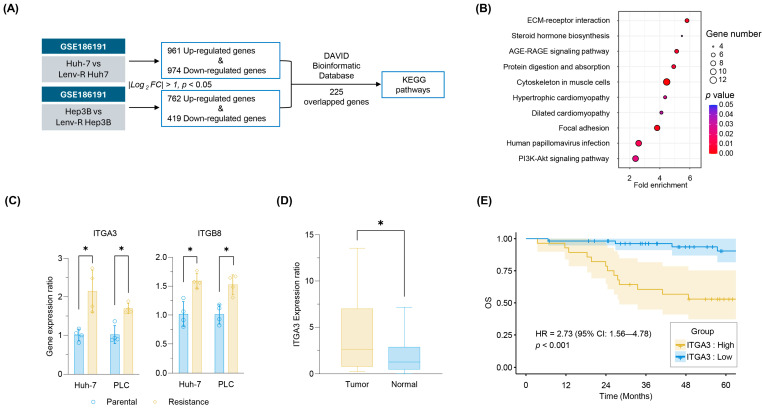
Gene Profiling Analysis Reveals Genes Involved in Lenvatinib Resistance. (**A**) Workflow showing the identification of differentially expressed genes from the GSE186191 dataset comparing parental and Lenvatinib-resistant Huh-7 and Hep3B cells. A total of 225 commonly altered genes were identified and analyzed using the DAVID Bioinformatics Database. (**B**) Pathway enrichment analysis of the 225 shared genes identified the top 10 KEGG pathways, with the ECM-receptor interaction pathway being the most enriched, including ITGA3 and ITGB8, which were also commonly found in the Focal Adhesion and PI3K/AKT signaling pathways. (**C**) qRT-PCR analysis of ITGA3 and ITGB8 expression in parental and rHuh-7 and rPLC cells. Both resistant cell lines exhibited significantly higher expression of these genes than their parental cell lines. (**D**) ITGA3 expression in 83 paired clinical samples (tumor tissue vs. adjacent non-tumor tissue). (**E**) Kaplan–Meier survival curve showing worse prognosis for patients with high ITGA3 expression (*p* < 0.001; HR = 2.73, 95% CI: 1.56–4.78). Data represent mean ± SD (* *p* < 0.05 vs. control).

A comprehensive genome-wide transcriptomic profiling analysis was performed on parental and Lenvatinib-resistant HCC cells using a public dataset (GSE186191) to identify the most important signaling and regulatory pathways associated with Lenvatinib resistance. This analysis identified 961 upregulated and 974 downregulated genes in Lenvatinib-resistant Huh-7 cells, and 762 upregulated and 419 downregulated genes in Lenvatinib-resistant Hep3B cells (|log2 FC| > 1.0, *p* < 0.05; Figure 4A). A total of 225 genes were found to be commonly altered in both cell lines, and pathway enrichment analysis using the Database for Annotation, Visualization, and Integrated Discovery (DAVID) Bioinformatics Database 2021 (https://david.ncifcrf.gov/, accessed on 22 October 2024) [23] revealed that 10 Kyoto Encyclopedia of Genes and Genomes (KEGG) pathways were preferentially enriched (FC > 2.0, *p* < 0.05; Figure 4B). The extracellular matrix (ECM)-related signaling pathway was identified as the most relevant pathway with the highest enrichment score. Additionally, other related pathways, such as the focal adhesion and PI3K-Akt signaling pathways, which are associated with proliferative signaling, were also enriched. Integrins, components of these pathways, have recently been shown to contribute to cancer malignancy, including chemotherapy resistance, by interacting with proliferative pathways such as the PI3K-AKT pathway and other signaling networks [24,25,26]. Based on these findings, we hypothesized that the ECM-related signaling pathway may play a crucial role in Lenvatinib resistance in hepatocellular carcinoma. To test this hypothesis, we examined the expression of ITGA3 and ITGB8, key components of this pathway, in our Lenvatinib-resistant cell lines. Both genes were significantly upregulated in resistant cells compared to parental controls (ITGA3: rHuh-7: FC = 2.1, *p* = 0.007; rPLC: FC = 1.7, *p* < 0.001; ITGB8: rHuh-7: FC = 1.6, *p* = 0.005; rPLC: FC = 1.5, *p* = 0.004; Figure 4C). Given its higher fold change, ITGA3 was selected as the primary target for subsequent experiments. Taken together, these findings indicate that ITGA3 is a key ECM-related factor contributing to Lenvatinib resistance in HCC.

To further validate the biological relevance of ITGA3 in clinical HCC, we analyzed ITGA3 expression in paired tumor and non-tumor mucosa specimens (*n* = 83). Quantitative reverse transcription polymerase chain reaction (qRT-PCR) revealed that ITGA3 expression was significantly upregulated in tumor tissues compared to adjacent non-tumor tissues (*p* < 0.001, Figure 4D). We then evaluated the prognostic impact of ITGA3 expression using overall survival (OS) data. Kaplan–Meier survival analysis demonstrated that patients with high ITGA3 expression had significantly poorer OS than those with low ITGA3 expression (*p* < 0.001; HR = 2.73, 95% CI: 1.56–4.78, Figure 4E). These findings support the notion that ITGA3 may serve as a prognostic biomarker and a potential therapeutic target in HCC.

### 2.5. siRNA-Mediated Knockdown of ITGA3 Suppresses Tumorigenic Features and Induces Anoikis Sensitivity in HCC Cells

**Figure 5:**
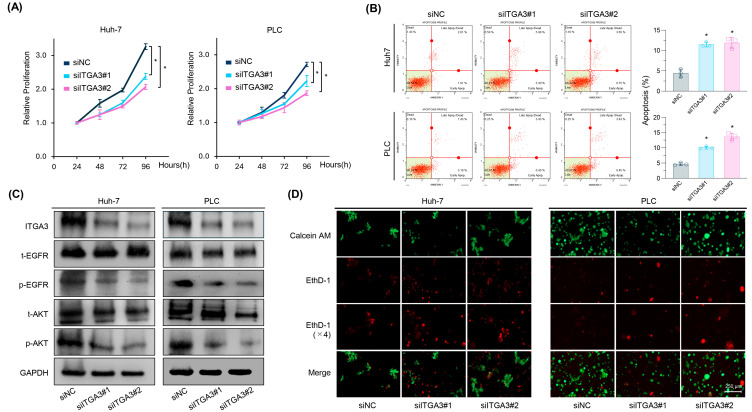
siRNA-mediated knockdown of ITGA3 suppresses tumorigenic phenotypes and induces apoptosis in HCC cell lines. (**A**) Proliferation assay after ITGA3 siRNA transfection. (**B**) Annexin V apoptosis assay in rHuh-7 and rPLC after ITGA3 knockdown. ITGA3 knockdown significantly increased apoptosis rates compared to control siRNA (siNC). (**C**) Western blot analysis showing decreased ITGA3 expression and inhibition of the EGFR–AKT signaling pathway following ITGA3 knockdown in rHuh-7 and rPLC. GAPDH was used as an internal control. (**D**) Representative images of anoikis assays in rHuh-7 and rPLC after ITGA3 knockdown. Calcein AM was used to stain viable cells (green), and EthD-1 was used to stain dead cells (red). Data represent mean ± SD (* *p* < 0.05 vs. control). (See also Figure S1 for functional assays demonstrating suppression of colony formation, migration, invasion, and stemness-related gene expression following ITGA3 knockdown).

To elucidate the role of ITGA3 in Lenvatinib resistance in HCC, we performed siRNA-mediated knockdown of ITGA3 in Lenvatinib-resistant HCC cell lines (rHuh-7 and rPLC). Combined ITGA3 knockdown and Lenvatinib treatment significantly inhibited cell proliferation in rHuh-7 (*p* < 0.001 for siRNA #1 and siRNA #2) and rPLC (*p* = 0.002 for siRNA #1 and *p* < 0.001 for siRNA #2 in both cell lines; Figure 5A). In addition to proliferation, we examined whether ITGA3 knockdown affected other malignant phenotypes of resistant HCC cells. ITGA3 knockdown markedly suppressed colony formation (Figure S1A), migration in wound-healing assays (Figure S1B), and invasion (Figure S1C), and also reduced the expression of stemness-related genes, including CD44, CD133, and OCT4 (Figure S1D). These findings indicate that ITGA3 regulates multiple aggressive characteristics of HCC cells.

To further evaluate the impact of ITGA3 knockdown on cell viability, apoptosis was quantified using Annexin V binding assays. Compared with cells transfected with negative control siRNA, ITGA3 knockdown significantly increased apoptosis in both rHuh-7 (FC = 2.58, *p* < 0.01 for siRNA #1; FC = 2.67, *p* < 0.01 for siRNA #2; Figure 5B) and rPLC (FC = 2.17, *p* < 0.01 for siRNA #1; FC = 2.93, *p* < 0.01 for siRNA #2; Figure 5B). Western blot analysis confirmed that ITGA3 knockdown markedly reduced ITGA3 protein expression and inhibited activation of the EGFR–AKT signaling pathway (Figure 5C). These findings suggest that ITGA3 is closely involved in the EGFR–AKT pathway, a crucial mediator of Lenvatinib resistance.

Anoikis, a form of programmed cell death triggered by detachment from the ECM, is frequently circumvented in cancer cells via dysregulation of signaling pathways such as Ras/ERK and PI3K/AKT, as well as through remodeling of the ECM [27,28,29]. Anoikis resistance is strongly associated with chemotherapy resistance and represents a critical therapeutic target. Integrins, including ITGA3, have been implicated in promoting anoikis resistance and metastatic potential by interacting with receptor tyrosine kinases and activating survival signaling pathways that drive malignant transformation [30,31,32,33,34]. We hypothesized that ITGA3-mediated anoikis resistance contributes to Lenvatinib resistance in HCC. To test this, we performed anoikis assays following ITGA3 knockdown in Lenvatinib-resistant cells. As expected, ITGA3 knockdown significantly reduced anoikis resistance (Figure 5D). Collectively, these findings demonstrate that ITGA3 plays a critical role in multiple malignant phenotypes of HCC cells, including proliferation, colony formation, migration, invasion, stemness, apoptosis, and anoikis resistance, primarily through the activation of the EGFR–AKT pathway. Thus, ITGA3 represents a promising therapeutic target for overcoming Lenvatinib resistance in HCC.

### 2.6. Combined OPCs and Lenvatinib Therapy Restores Anoikis Sensitivity Through ITGA3–EGFR–AKT Pathway Inhibition

**Figure 6:**
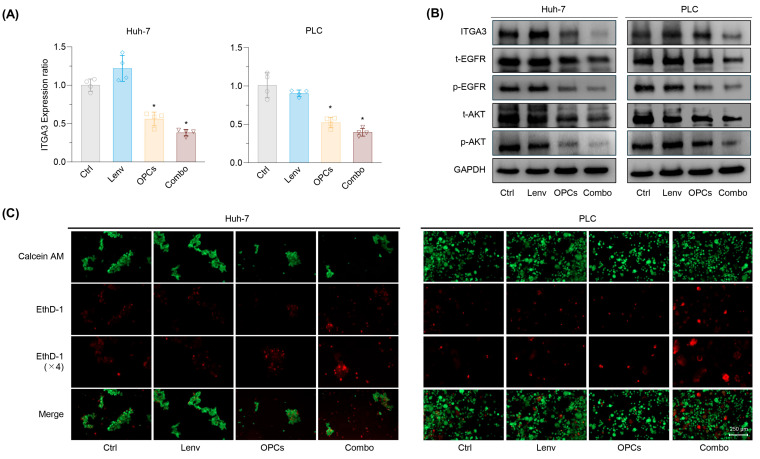
OPCs restore anoikis sensitivity and overcome Lenvatinib resistance by targeting ITGA3 and inhibiting EGFR–AKT signaling. (**A**) qRT-PCR analysis of ITGA3 expression in rHuh-7 and rPLC cell lines treated with Lenv, OPCs, or the combination of both. OPCs treatment significantly downregulated ITGA3 expression at the mRNA level. (**B**) Western blot analysis showing the effect of treatments on ITGA3 expression and EGFR–AKT signaling pathway activation in rHuh-7 and rPLC. OPCs and the combination treatment suppressed ITGA3 and the phosphorylation of EGFR, AKT. **(C)** Representative images from anoikis assays in rHuh-7 and rPLC treated with control, Lenvatinib, OPCs, or the combination. Calcein AM was used to stain viable cells (green), and EthD-1 was used to stain dead cells (red). Combination therapy restored anoikis sensitivity in both cell lines. Data represent mean ± SD (* *p* < 0.05 vs. control).

OPCs, widely studied for their anti-tumor properties, are known to inhibit multiple signaling pathways, thereby suppressing tumor growth, migration, and invasion [35,36,37,38]. Based on these characteristics, we hypothesized that OPCs may overcome Lenvatinib resistance by targeting the ITGA3–EGFR–AKT signaling axis and promoting anoikis. To test this hypothesis, we evaluated mRNA and protein expression levels of the ITGA3-EGFR–AKT signaling axis following OPCs treatment. In Lenvatinib-resistant rHuh-7 and rPLC, Lenvatinib monotherapy had a minimal effect on ITGA3 mRNA and protein levels (Figure 6A,B). In contrast, OPC treatment significantly downregulated ITGA3 expression. Notably, the combination of OPCs and Lenvatinib further suppressed ITGA3 expression compared to either treatment alone, accompanied by a marked reduction in EGFR and AKT phosphorylation (Figure 6A,B). Additionally, anoikis assays demonstrated that the combination therapy significantly restored anoikis sensitivity (Figure 6C), further supporting the role of ITGA3 in mediating anoikis resistance and its contribution to Lenvatinib resistance. These findings indicate that OPCs-mediated suppression of ITGA3 plays a central role in restoring Lenvatinib sensitivity in resistant HCC cells. Collectively, these results suggest that targeting ITGA3 through the combined use of OPCs and Lenvatinib represents a promising therapeutic strategy for overcoming Lenvatinib resistance in HCC.

## 3. Discussion

HCC remains a major global health concern, representing the most common primary liver malignancy and the sixth most frequently diagnosed cancer worldwide [1]. While curative options such as surgical resection and liver transplantation are available for early-stage HCC, advanced cases typically require systemic therapies. Currently, Lenvatinib and the combination of Atezolizumab plus Bevacizumab are approved as first-line treatments [5,39]. Lenvatinib, a multi-tyrosine kinase inhibitor, is particularly effective in blocking several key growth factor receptors, including vascular endothelial growth factors and fibroblast growth factor receptors [40]. However, despite its clinical efficacy, the objective response rate of Lenvatinib is limited to approximately 24%, and the development of drug resistance is a significant clinical challenge [4]. The mechanisms underlying Lenvatinib resistance are complex and multifactorial. Our transcriptome analysis revealed that ECM-related signaling pathways, including the expression of ITGA3, were upregulated in Lenvatinib-resistant cells. In particular, integrins are known to mediate cell-ECM interactions and have been shown to play important roles in tumor progression, metastasis, anoikis resistance, and chemotherapy resistance through activation of downstream pathways such as the EGFR–AKT pathway [24,25,26,27,28,30,32].

Natural compounds with broad-spectrum anti-cancer activities, such as OPCs, offer a promising adjunctive approach. OPCs, naturally occurring polyphenols found in various fruits, vegetables, and plants, have demonstrated potent anti-tumor effects in a variety of cancers [21]. These effects are mediated through the modulation of several oncogenic signaling pathways, including growth signaling pathways, drug transporter systems, and metabolic pathways such as protein export, glutathione metabolism, and porphyrin metabolism [19,41,42]. The unique properties of OPCs, including their favorable safety profile and ability to target multiple pathways simultaneously, make them attractive candidates for combination therapy in cancer treatment.

In this study, we hypothesized that overexpression of ITGA3 activates the EGFR–AKT pathway through enhanced ECM pathways, contributing to Lenvatinib resistance in HCC. We further proposed that OPCs could overcome this resistance by targeting the ITGA3–EGFR–AKT signaling axis. Our results demonstrated that OPC treatment, particularly in combination with Lenvatinib, effectively inhibited proliferation and invasion in Lenvatinib-resistant HCC cells. Mechanistically, OPCs significantly downregulated the ITGA3–EGFR–AKT axis. We also observed that OPCs treatment reduced the spheroid-forming capacity of resistant cells, suggesting that it may suppress cancer stem cell-like properties. Moreover, ITGA3 overexpression was found to contribute to anoikis resistance, a known mechanism of chemoresistance. Importantly, co-administration of OPCs and Lenvatinib enhanced anoikis sensitivity and restored drug responsiveness in resistant cells. Apoptosis assays further confirmed that the combination therapy induced cell death, supporting its therapeutic potential.

We would like to acknowledge some of the limitations of this study. First, the current study employed only two Lenvatinib-resistant cell lines, which may limit the generalizability of the findings. Validation in a broader panel of HCC models is necessary. Second, although our results provide strong in vitro evidence, preclinical studies in animal models are essential to assess the in vivo efficacy and safety of the OPCs and Lenvatinib combination therapy. Nevertheless, this study is the first to demonstrate that ITGA3 plays a critical role in Lenvatinib resistance in HCC and that OPCs can potentially overcome this resistance by targeting the ITGA3–EGFR–AKT pathway. Moreover, our clinical sample analysis revealed a significant association between high ITGA3 expression and poor prognosis in HCC patients, further supporting ITGA3 as a promising and clinically relevant therapeutic target. Future studies should focus on validating these findings in vivo and exploring the therapeutic potential of OPCs in preclinical animal models.

## 4. Materials and Methods

### 4.1. Patient Cohort

This study included frozen tumor and normal tissue specimens from 83 HCC patients enrolled at Tokushima University, Japan. Patient characteristics are detailed in Table S1. All patients underwent surgical resection, and tumors were histologically confirmed as HCC. Tumors were classified according to the Union for International Cancer Control (UICC) TNM Classification of Malignant Tumors, version 7. The study was conducted in accordance with the ethical principles outlined in the Declaration of Helsinki, as revised in 2024, and was approved by the ethics committees of the participating institutions. Written informed consent was obtained from all patients prior to inclusion in the study.

### 4.2. Cell Culture and Materials

Two human HCC cell lines, Huh-7 and PLC-PRF-5 (PLC), were examined in this study and obtained from the RIKEN BioResource Center Cell Bank (Tsukuba, Japan). Both cell lines were cultured in Dulbecco’s Modified Eagle’s Medium (DMEM; Gibco, Carlsbad, CA, USA) containing 10% fetal bovine serum, and 1% penicillin and streptomycin, maintained in a humidified incubator at 37 °C in 5% CO_2_.

### 4.3. Herbal Preparations and Drug Preparation

The OPCs used in this study were obtained from VX1^®^, a French grape seed extract (EuroPharma USA, Green Bay, WI, USA), standardized by the manufacturer to contain ≥99% total polyphenols and ≥80% low-molecular-weight OPCs (monomers, dimers, and trimers). The powder was dissolved in dimethyl sulfoxide (DMSO; Sigma-Aldrich, St. Louis, MO, USA) and further diluted in complete culture medium to obtain working concentrations for in vitro experiments.

Lenvatinib (Sigma-Aldrich) was dissolved in DMSO (Sigma-Aldrich). The stock solution of Lenvatinib was carefully stored at −20 °C in the dark, ensuring its stability and reliability. This stock solution was diluted with a complete culture medium to the necessary experimental concentrations before each application.

### 4.4. Cell Viability Assays

For these assays, 5 × 10^3^ cells per well were seeded into 96-well plates under each culture condition. After overnight incubation for cell adherence to the plates, all cell lines were treated for 48h with Lenvatinib, OPCs, or their combination. Following completion of the treatments, Cell Counting Kit-8 (CCK-8) assays (Dojindo Molecular Technologies, Inc., Kumamoto, Japan; Cat# CK04) were performed according to the manufacturer’s instructions. Synergy scores were calculated using SynergyFinder 3.0 (https://synergyfinder.fimm.fi), a freely available tool for interactive analysis and visualization of combination response outcomes [43].

### 4.5. Establishment of Lenvatinib-Resistant HCC Cell Lines

Lenvatinib-resistant cells were induced from continuous culture of Huh-7 and PLC cell lines in Lenvatinib-enriched medium. The parental cell lines were treated with increasing doses of Lenvatinib, with concentrations of 0.5 µM administered weekly over 10 months. Finally, resistant cells that could grow stably in medium containing 20 µM Lenvatinib were selected for further experiments.

### 4.6. Invasion Assay

For the invasion assay, cells (2.5 × 10^4^ cells) treated with Lenvatinib, OPCs, and their combination for 48 h were grown in 24-well transwell chambers (8-μm pore size) coated with Matrigel (BD Biosciences, Franklin Lakes, NJ, USA). After 48 h, the invaded cells were fixed and stained with Diff-Quick (Thermo Fisher Scientific, Waltham, MA, USA) and then counted.

### 4.7. The Wound Healing Assay

For the wound healing assay, cells treated with Lenvatinib, OPCs, and their combination were seeded into 6-well plates and grown to 80% confluency. Wounds were made by scraping 200 µL of monolayer cells with a pipette tip (RAININ, LLC, Oakland, CA, USA). 24 h after wounding, treated and control cells were examined under a microscope (Zeiss, Oberkochen, Germany).

### 4.8. The Colony Formation Assay

For the colony formation assay, 5 × 10^2^ cells treated with Lenvatinib, OPCs, and their combinations for 48 h were seeded into 6-well plates (Corning, Tehama County, CA, USA). Seven to ten days later, the number of colonies with 50 or more cells was counted, and relative changes were measured.

### 4.9. Apoptosis Assay

For the Annexin V binding apoptosis assay, cells were seeded in 6-well plates and then treated with Lenvatinib, OPCs, and their combinations for 48 h. Apoptotic cell fractions were then measured using the Muse Cell Analyzer (Millipore Corp, Billerica, MA, USA) and Annexin V and Dead Cell Kit (Luminex Corp, Austin, TX, USA) according to the manufacturer’s instructions.

### 4.10. Spheroid Formation Assays

Methods for these assays have been described previously [44]. Briefly, Lenvatinib-resistant cell lines were seeded in serum-free DMEM-F12 medium (STEMCELL Technologies, Vancouver, BC, Canada) in ultra-low adherent plates (Corning); spheroids were formed over 5–10 days, and spheroid size and number were counted using ImageJ software (version 1.53; National Institutes of Health, Bethesda, MD, USA).

### 4.11. Identification of Differentially Expressed Genes and Functional Pathway Enrichment in Lenvatinib-Resistant HCC Cells

To identify genes that are differentially regulated in Lenvatinib-resistant HCC cells, we analyzed gene expression data obtained from the Gene Expression Omnibus (GEO) database (https://www.ncbi.nlm.nih.gov/geo/, accessed on 22 October 2024; GSE186191). Gene expression profiles of Lenv-R HCC cell lines were compared with those of parental HCC cell lines. Differentially regulated genes were defined by an FC > 1.0 or <−1.0 and a *p*-value < 0.05. Pathway enrichment analysis was conducted using the KEGG pathway analysis tool (https://david.ncifcrf.gov/, accessed on 22 October 2024). Enriched pathways were identified using the DAVID Bioinformatics Resources 2021 (https://david.ncifcrf.gov/, accessed on 22 October 2024) [23], with enrichment criteria of fold enrichment > 2.0 and *p* < 0.05. Scatter plots and heatmaps of the differentially regulated genes were generated based on the results of the enrichment analysis.

### 4.12. RNA Extraction and qRT-PCR

Total RNA was isolated from cancer cells treated with Lenvatinib, OPCs, or their combination for 24 h using the Qiagen miRNeasy Kit (Qiagen, Hilden, Germany). Complementary DNA (cDNA) was synthesized from the extracted RNA using a high-capacity cDNA reverse transcription kit (Thermo Fisher Scientific), QuantStudio 6/7 Flex RT-PCR System (Applied Biosystems, Foster City, CA, USA), and qRT-PCR was performed using SensiFAST SYBR Lo-ROX Kit (Bioline, London, UK). The housekeeping gene GAPDH was used as an internal control. Gene expression levels were quantified using the ΔCt method, which calculates the difference in Ct values between the target and control genes. The primer sequences used in this study are described in Table S2.

### 4.13. Protein Isolation and Western Blotting

Western blotting was performed according to previous studies [44,45]. Total protein was extracted from HCC cell lines treated with Lenvatinib, OPCs, or a combination of the two for 48 h. Cells were collected using a plastic scraper and washed three times with cold Phosphate-buffered saline (PBS). Cells were then lysed in ice-cold RIPA protein extract containing a protease inhibitor cocktail (Thermo Fisher Scientific). Extracted proteins were denatured in 4× Laemmli sample buffer (BIO-RAD, Hercules, CA, USA), which contains 5% 2-mercaptoethanol. Protein concentrations were determined using the BCA method (Thermo Fisher Scientific). Equal amounts of protein samples (60 μg) were separated by SDS-PAGE using 7.5% or 10% Mini-PROTEAN TGXTM Precast Gels (BIO-RAD), transferred to nitrocellulose membranes, and then transferred to 0.45 μm. The membranes were blocked with 5% bovine serum albumin (Sigma-Aldrich) in Tris-buffered saline (BIO-RAD) supplemented with 0.1% Tween-20 (Sigma-Aldrich) for 1 h at room temperature. Membranes were incubated overnight at 4 °C with the following primary antibodies: GAPDH (1:5000, Proteintech, Cat# 10494-1-AP), PARP (1:1000, CST, Cat# 9532S), Cleaved-PARP (1:1000, CST, Cat# 5625S), Caspase-3 (1:1000, CST, Cat# 9662S), Cleaved-Caspase-3 (1:1000, CST, Cat# 9661S), Bax (1:1000, CST, Cat# 41162), CD44 (1:1000, CST, Cat# 3570S), CD133 (1:1000, Merck Millipore, Cat# MAB4399), OCT4 (1:1000, CST, Cat# 75463S), ITGA3 (1:1000, Proteintech, Cat# 66070-1-Ig), EGFR (1:1000, CST, Cat# 4267T), Phospho-EGFR (Ser473, 1:1000, CST, Cat# 3777S), AKT (1:1000, CST, Cat# 9272), and Phospho-AKT (Ser473, 1:1000, CST, Cat# 4060S). After washing, membranes were incubated with HRP-conjugated secondary antibodies (CST) for 1 h at room temperature. Protein bands were visualized using an HRP-based chemiluminescence kit (Thermo Fisher Scientific) and imaged with Gel Imaging Systems (BIO-RAD). GAPDH was used as a loading control.

### 4.14. Transfection of Small Interfering RNA

In this study, 1 × 10^6^ cells were reverse-transfected using Lipofectamine RNAiMAX (Thermo Fisher Scientific) with either ITGA3-specific siRNA (Cat. No. 2012881; Assay IDs: 107727 and 16478) or a negative control siRNA and subsequently plated in 6–cm dishes. After 48 h of transfection, the cells were utilized for RNA and protein extraction as well as additional assays.

### 4.15. Anoikis Assay

Anoikis resistance was assessed using the CytoSelect™ 96-Well Anoikis Assay (Cell Biolabs, San Diego, CA, USA; Cat# CBA-081). Huh-7 and PLC HCC cells were seeded at a density of 2 × 10^4^ cells per well and cultured for 24 h at 37 °C in a 5% CO_2_ atmosphere. For fluorometric detection, cells were stained with Calcein-AM (Invitrogen, Part No. 108002, 500X) and Ethidium Homodimer-1 (EthD-1; Invitrogen, Part No. 108003, 500X) diluted to 100X in culture medium. Live and dead cells were detected by fluorescence microscopy (Calcein-AM: Ex 485 nm/Em 515 nm; EthD-1: Ex 525 nm/Em 590 nm) and quantified using a fluorescence microplate reader (Tecan Trading AG, Männedorf, Switzerland).

### 4.16. Statistical Analysis

All experiments were conducted in triplicate, and the results were presented as mean ± standard deviation (SD). For statistical comparisons between two groups, the Wilcoxon rank-sum test (Mann–Whitney U test) was applied. A *p*-value of less than 0.05 was considered statistically significant. Kaplan–Meier survival curves were generated to evaluate OS, and differences between groups were assessed using the log-rank test. In addition, univariate Cox proportional hazards regression analysis was performed to calculate hazard ratios (HR) and 95% confidence intervals (CI) for each group. All statistical analyses, including survival analysis and HR calculation, were performed using R software (version 4.3.1), utilizing the survival and survminer packages.

## 5. Conclusions

In conclusion, our findings suggest that ITGA3-mediated activation of the EGFR–AKT pathway and associated anoikis resistance play critical roles in Lenvatinib resistance. Targeting this axis with OPCs restores anoikis sensitivity and resensitizes resistant HCC cells to Lenvatinib. These results highlight a novel therapeutic avenue for overcoming Lenvatinib resistance and provide a promising rationale for future clinical translation of OPCs-based combination strategies in HCC treatment.

## Data Availability

The datasets generated and/or analyzed during the current study are available from the corresponding author on reasonable request.

## Supplementary Materials

The following supporting information can be downloaded at: https://www.mdpi.com/article/10.3390/ph18091361/s1, Figure S1: Functional assays showing suppression of colony formation, migration, invasion, and stemness-related gene expression following ITGA3 knockdown; Figures S2–S5: Raw data of Western Blot; Table S1: Clinicopathological variables of HCC patients; Table S2: List of primers for RT-qPCR used in this study; Table S3: List of primary antibodies for Western blotting.

## Abbreviations

The following abbreviations are used in this manuscript:

HCC	Hepatocellular carcinoma
ICIs	Immune checkpoint inhibitors
TKIs	Multi-tyrosine kinase inhibitors
OS	Overall survival
IC_50_	Half-maximal inhibitory concentration
OPCs	Oligomeric proanthocyanidins
PLC	PLC-PRF-5
FC	Fold change
ECM	Extracellular matrix
EGFR	Epidermal growth factor receptor
DMEM	Dulbecco’s Modified Eagle’s medium
DMSO	Dimethyl sulfoxide
FBS	Fetal bovine serum
DAVID	Database for Annotation, Visualization, and Integrated Discovery
KEGG	Kyoto Encyclopedia of Genes and Genomes
CCK-8	Cell Counting Kit-8
PBS	Phosphate-buffered saline
SD	Standard deviation
HR	Hazard ratios
CI	Confidence intervals
cDNA	Complementary DNA
qRT-PCR	Quantitative reverse transcription polymerase chain reaction
