# Supplementary material for: Oligomeric Proanthocyanidins Reverse Lenvatinib Resistance in Hepatocellular Carcinoma Through ITGA3-Mediated Pathway
Source: Pharmaceuticals (Basel). 2025 Sep 12;18(9):1361. doi: 10.3390/ph18091361 (PMC12472469; doi:10.3390/ph18091361)
Supplement: Supplementary file 1 [file pharmaceuticals-18-01361-s001.zip › pharmaceuticals-3830018-supplementary.pdf]

Supplementary Materials:

Figure S1: Knockdown of ITGA3 suppresses colony formation, migration, invasion, and stemness-related gene expression in HCC cells. (**A**) Colony formation assays showing the number of colonies in Huh7 and PLC cells transfected with negative control siRNA (siNC) or ITGA3-targeting siRNAs (siITGA3#1, siITGA3#2). Quantification is shown on the right. (**B**) Wound healing assays assessing cell migration in Huh7 and PLC cells at 0 h and 24 h post-scratch. Relative migration rates are quantified on the right. (**C**) Transwell invasion assays in Huh7 and PLC cells transfected with siNC or siITGA3 siRNAs. Representative images (left) and quantification of invasive cells (right) are shown. (**D**) Relative mRNA expression levels of stemness-related markers (CD44, CD133, OCT4) in Huh7 and PLC cells after ITGA3 knockdown, determined by qRT-PCR.

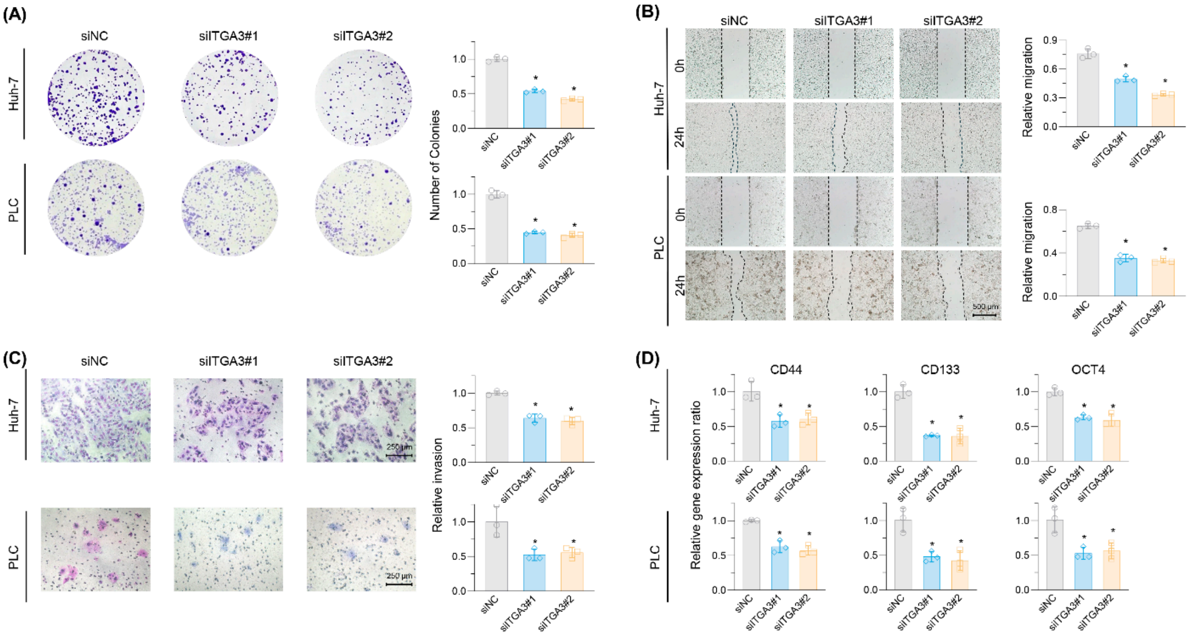

Figure S2: Original data of Western Blot

Figure3B

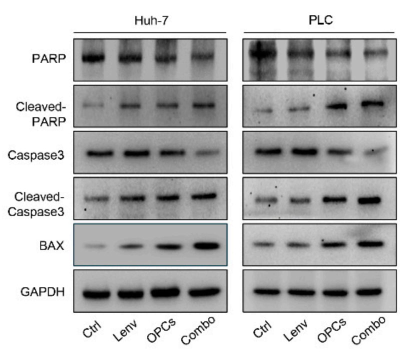

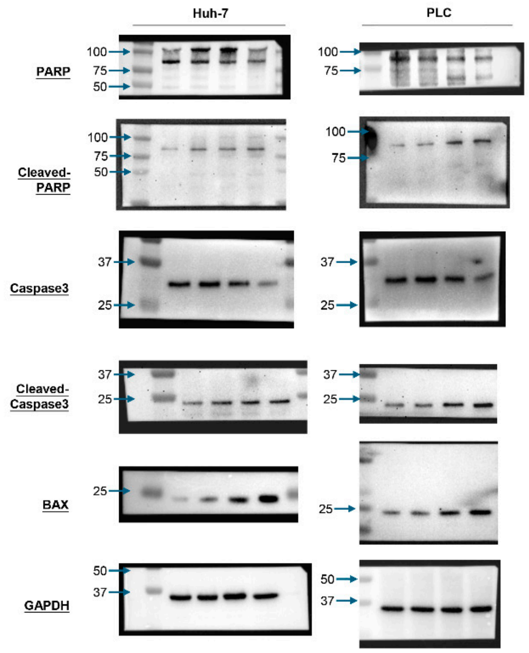

Figure S3: Original data of Western Blot

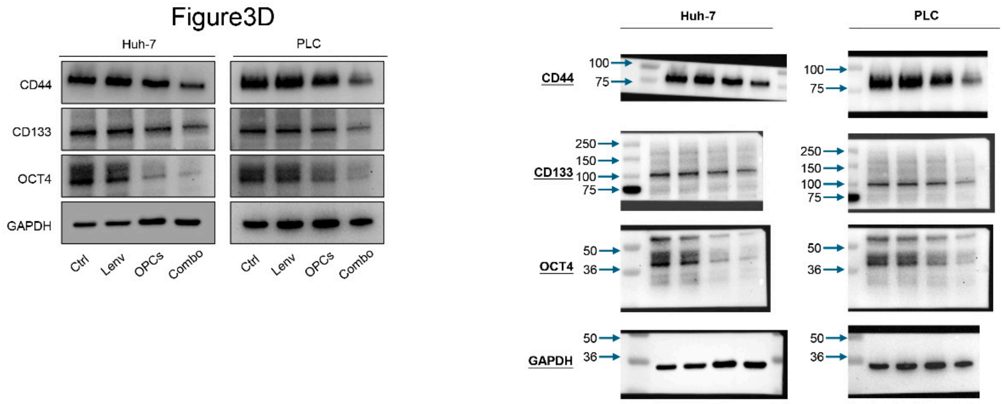

Figure S4: Original data of Western Blot

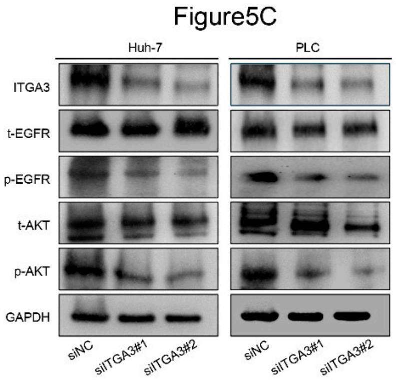

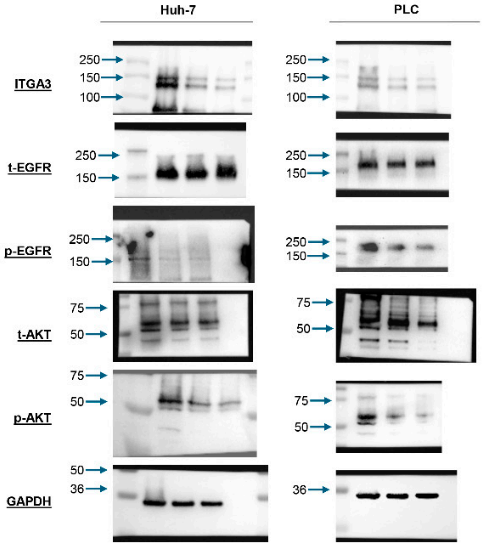

Figure S5: Original data of Western Blot

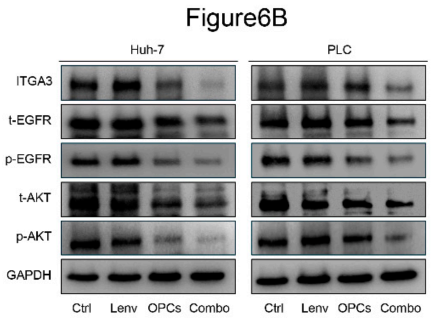

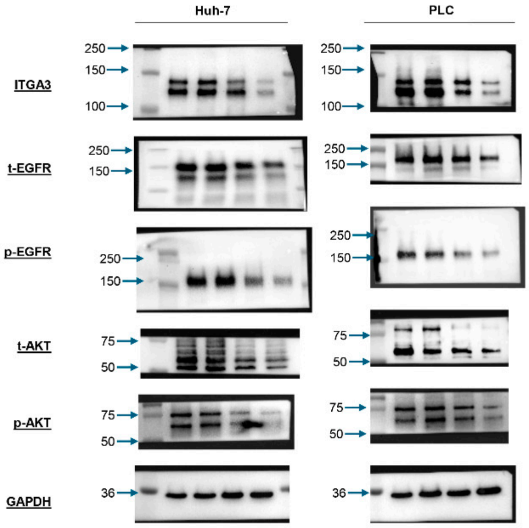

Table S1. Clinicopathological variables of hepatocellular carcinoma patients

Characteristics	Clinical cohort (n=83)
**Age (years)**	
≤60	11
>60	72
**Gender**	
Male	63
Female	20
**Differentiation**	
Well	14
Moderate	61
Poor	8
**Virus**	
HBV	10
HCV	29
NBNC	44
**Tumor Number**	
Single	81
Multiple	2
**Tumor Size (cm)**	
≤5	61
>5	22
**AFP (ng/mL)**	
≤400	79
>400	4
**Cirrhosis**	
No	64
Yes	19
**AJCC Stage**	
I	17
II	66
**Diabetes**	
Present	48
Absent	35

Table S2. List of primers for RT-qPCR used in this study.

Name	Forward	Reverse
ITGA3	TGTGGCTTGGAGTGACTGTG	TCATTGCCTCGCACGTAGC
ITGB8	GTGAAAGTCATATCGGATGGCG	GCTATCAAGAGCGAGATGAGACG
CD44	CAATAGCACCTTGCCCACAAT	AATCACCACGTGCCCTTCTATGG
CD133	CAGAGTACAACGCCAAACCA	AAATCACGATGAGGGTCAGC
OCT4	CTGGGTTGATCCTCGGACCT	CCATCGGAGTTGCTCTCCA
GAPDH	TGAACGGGAAGCTCACTG	TCCACCACCCTGTTGCTGTA

Note: The primers of mRNAs were purchased from IDT, Coralville, Iowa, USA

Table S3. List of primary antibodies for western blotting

Target	Dilution ratio	Supplier	Catalogue Number
GAPDH	1:5000	Proteintech	10494-1-AP
PARP	1:1000	CST	9532S
Cleaved-PARP	1:1000	CST	5625S
Caspase3	1:1000	CST	9662s
Cleaved-caspase3	1:1000	CST	9661s
Bax	1:1000	CST	41162
CD44	1:1000	CST	3570S
CD133	1:1000	Merck Millipore	MAB4399
OCT4	1:1000	CST	75463S
ITGA3	1:1000	Proteintech	66070-I-Ig
EGF-Receptor	1:1000	CST	4267T
Phospho-EGF Receptor (Ser473)	1:1000	CST	3777S
AKT	1:1000	CST	9272
Phospho-Akt (Ser473)	1:1000	CST	4060S

Note: CST: Cell Signaling Technology
